# A Taiwanese food frequency questionnaire correlates with plasma docosahexaenoic acid but not with plasma eicosapentaenoic acid levels: questionnaires and plasma biomarkers

**DOI:** 10.1186/1471-2288-13-23

**Published:** 2013-02-16

**Authors:** Kuo-Liong Chien, Meei-Shyuan Lee, Yi-Tsen Tsai, Pey-Rong Chen, Hung-Ju Lin, Hsiu-Ching Hsu, Yuan-The Lee, Ming-Fong Chen

**Affiliations:** 1 Institute of Epidemiology and Preventive Medicine, College of Public School, National Taiwan University, Taipei, Taiwan; 2 Department of Internal Medicine, National Taiwan University Hospital, Taipei, Taiwan; 3 School of Public Health, National Defense Medical Center, Taipei, Taiwan; 4 Department of Dietetics, National Taiwan University Hospital, Taipei, Taiwan; 5 Chinese Medical University Hospital, Taichung, Taiwan

**Keywords:** N-3 fatty acid, Biomarker, Food frequency questionnaire

## Abstract

**Background:**

Little evidence is available for the validity of dietary fish and polyunsaturated fatty acid intake derived from interviewer-administered questionnaires and plasma docosahexaenoic acid (DHA) and eicosapentaenoic acid (EPA) concentration.

**Methods:**

We estimated the correlation of DHA and EPA intake from both questionnaires and biochemical measurements. Ethnic Chinese adults with a mean (± SD) age of 59.8 (±12.8) years (n = 297) (47% women) who completed a 38-item semi-quantitative food-frequency questionnaire and provided a plasma sample were enrolled. Plasma fatty acids were analyzed by capillary gas chromatography.

**Results:**

The Spearmen rank correlation coefficients between the intake of various types of fish and marine n-3 fatty acids as well as plasma DHA were significant, ranging from 0.20 to 0.33 (*P* < 0.001). In addition, dietary EPA, C22:5 n-3 and DHA were significantly correlated with the levels of marine n-3 fatty acids and DHA, with the Spearman rank correlation coefficients ranging from 0.26 to 0.35 (*P* < 0.001). Moreover, compared with those in the lowest fish intake quintile, participants in the highest quintile had a significantly higher DHA level (adjusted mean difference, 0.99 ± 0.10%, test for trend, *P* < 0.001). Similar patterns between dietary DHA intake and plasma DHA levels were found. However, the association between dietary fish intake and plasma EPA was not significant (test for trend, *P* = 0.69).

**Conclusions:**

The dietary intakes of fish and of long chain n-3 fatty acids, as determined by the food frequency questionnaire, were correlated with the percentages of these fatty acids in plasma, and in particular with plasma DHA. Plasma DHA levels were correlated to dietary intake of long-chain n-3 fatty acids.

## Background

Dietary eicosapentaenoic acid (EPA) and docosahexaenoic acid (DHA) intake from marine fish is associated with cardiovascular disease prevention [1]. In addition, nutrient assessment instruments have improved our understanding of the causal factors of disease and are used in clinical and counseling situations, and in public policy decisions and recommendations. Although nutrient assessments of EPA and DHA intake were available in Caucasian [2-4], a tailored food frequency questionnaire (FFQ) to assess food intake in Taiwan is lacking.

Interviewer-administered semi-quantitative FFQs are considered to be the most feasible method for use in large-scale epidemiological research, and the validation of dietary EPA and DHA intake has been examined in an older population [5], for lipid concentration [6], and for coronary heart disease risk. However, validation of FFQ performance to determine how well an FFQ estimates nutrient intake as compared with diet records or repeated 24-hour recalls was affected by the correlated recall errors [7,8].

The measurement of plasma fatty acids by gas chromatography provides useful measurements of EPA and DHA, especially in high-throughput technology [9,10]. Furthermore, the errors of the dietary intake measurements from FFQs and serum biomarkers are independent, so it is feasible to evaluate the correlation of an FFQ in assessing EPA, DHA and other fatty acid intake in a clinical setting. In addition, the measured EPA and DHA concentrations are related to dietary intake and they are time-integrated, so it is feasible to use serum concentrations as the correlation tool for the reported FFQ [11].

Few FFQs have been developed specifically for use in ethnic Chinese populations [12], although literature about fish intake and n-3 fatty acids were available among European populations [13-15]. In addition, validation for the performance of FFQs using biochemical indicators of EPA and DHA intake have been reported in various populations[16-21]; however, the data for ethnic Chinese was scanty.

The validity of nutrient intake using an interviewer-administered FFQ was tested among young adults in Taiwan [12], and FFQs have been shown to be feasible for association studies between dietary intake and disease risk [12,22-24]. Therefore, we aimed to investigate the validity of an interviewer-administered FFQ and biochemical indicators of plasma marine fatty acids among Taiwanese in various clinical settings.

## Methods

### Study population

We conducted this cross-sectional study during 2009 and collected the participants from various settings, including patients from the outpatient clinic and health examination station at National Taiwan University Hospital, and a clinic in the local hospital in Chin-Shan community, where we have followed a cohort since 1990 [25-27]. We used the same protocol to recruit participants from the outpatient settings for the semi-quantitative food frequency questionnaire and blood sampling as the standard procedure, and we have conducted a dietary assessment study in the same setting [28].

Blood pressure was measured by trained medical assistants in a resting position. Body mass index (BMI) was calculated as weight (in kilograms)/height (in meters)^2^. Waist circumference was measured according to the standard procedure [25,29].

### Dietary assessment

A 38-item Chinese FFQ, combined with open questions on cooking oils most frequently consumed, was administered by an interviewer to estimate dietary intake over the preceding year; it was a shortened version of a validated FFQ for Taiwanese [12]. In brief, this FFQ was designed to assess usual dietary intake over a 1-year period. Each participant was asked the frequency with which he/she ate a certain amount of each specific food. Photographs of foods, showing different portion sizes, were used to facilitate quantification of intake. Major contributor foods and predictors for fat, protein, carbohydrate, vitamin A, vitamin C and calcium and n-3 fatty acids were included [12]. Food items were arranged into sections of the major food groups; dairy, eggs, meat, poultry, fish, seafood, organ meat, soybean products, vegetables, fruits, staples, sugary drinks, pickles, sodium containing condiments, cooking methods, cooking oils, and use of supplements. Similar foods were listed close to each other to prevent redundant recollection. The frequency response section included seven categories; “never or less than once per month”, “1 to 3 times per month”, “once per week”, “2 to 4 times per week”, “5 to 6 times per week” “once per day”, “twice or more per day”. Tea intake and alcoholic drinks were included in the FFQ.

Dietary intake estimates for various fatty acids were derived from the FFQ by summing the product of nutrient content of each food item derived from a previously established nutrient database [30,31], portion size, and frequency of consumption. The food-composition database used to calculate nutrient values was based primarily on the Taiwan Food Composition Data Base [32-34] and other published data resources [22,23,35].

With regards to the intake of fish and seafood, the questionnaire included three questions on fish and seafood consumption (Additional file 1): “deep sea fishes, such as codfish and salmon”; “other fishes, including farmed and fresh water fishes, such as mouthbreeder, hair-tail fish or mackerel pike (samba fish)”; and “seafood, such as shrimps, oysters, clams and cuttlefish”. Information on the use of either cod-liver oil or n-3 fatty acid supplements was obtained as part of the FFQ in the form of yes/no questions.

### Laboratory examination of lipids

Blood samples were sent to the core laboratory of the Department of Internal Medicine, National Taiwan University Hospital. The procedures for blood sampling and analytic methods have been described previously [25]. In brief, blood samples were collected from each participant after fasting for at least 12 hours. Serum total cholesterol levels were measured using the CHOD-PAP method (Boehringer Mannheim, Germany). HDL-C was measured following precipitation of apolipoprotein B-containing lipoproteins with phosphotungstic acid and magnesium ions (Boehringer Mannheim, Germany) [36]. Triglyceride concentrations were measured by the GPO-DAOS method (Wako Co., Japan). All of the lipids were measured using a Hitachi 7450 automated analyzer (Hitachi, Japan). LDL-C concentrations were calculated using the Friedewald formula [37]. The coefficient of variation was 5%.

### Measurements of plasma fatty acids

A 10-ml tube of EDTA-anticoagulated blood was collected, refrigerated at the site centers, and sent back within 3 hours to the National Taiwan University Hospital core laboratory. The blood was centrifuged at 800 × *g* for 10 min, then plasma was separated and dispensed into several aliquots and frozen at -70°C for analysis for fatty acid (FA) content by a single technician. After thawing, 0.5 mL of plasma was extracted with 0.5 mL methanol followed by 1.0 mL chloroform under a nitrogen atmosphere, and the lipid extract was filtered to remove protein. The methyl esters were then separated and measured on a 5890 gas chromatograph (Hewlett Packard, Avondale, PA) equipped with a 30 m-FFAT WCOT glass capillary column (J & W Scientific, Folsom, CA) and a flame-ionization detector. The identities of individual fatty acid peaks were ascertained by comparing each peak’s retention time to the retention times of known standards. The relative amount of each component (% of total fatty acids) was quantified by integrating the area under the peak, and dividing the result by the total area for all fatty acids.

### Statistical analysis

All data were presented as mean ± standard deviation for continuous variables and frequency for categorical variables. Means and standard deviations of various nutrients, including EPA and DHA, were calculated from total nutrient intake from the questionnaires. Fish and seafood were summarized as one entity from the FFQ. In addition, in calculating correlation coefficients, we expressed dietary fatty acid intake as a percentage of total fat intake to correspond with the measurement of fatty acids in plasma [18,38].

The total energy intake variables were examined for outliers, and erroneous values were corrected if possible and deleted if not [39]. To ensure the results were not sensitive to these values, analyses were repeated with and without outliers and the results were not obviously different. We estimated the partial Spearman correlation coefficient, after adjusting for gender, age, and total energy intake, between various intakes derived from the FFQ and plasma concentrations [2]. In addition, kappa statistics were applied to estimate the consistency between intakes derived from the FFQ and plasma measurement [40]. We adjusted gender, age, and total energy intake when we tested the association between fish, EPA and DHA concentrations. To further evaluate the associations between the biochemical indicators and FFQ-estimated intakes, we compared the mean blood values of DHA and EPA concentrations across quintiles of fish and DHA intake. We also calculated the mean blood levels of fatty acids for the quintiles of fish intake and compared levels between these extremes of intake, and used linear regression models to estimate the change in blood value as a function of FFQ intake, adjusted for the set of confounders noted above [41]. In addition, we used the Bland-Altman plot to check the potential systematic bias between biomarker and intake of FFQ between biomarker and intake of FFQ [20]. All analyses were performed with SAS software, Version 9.1 (SAS Institute, Cary, NC).

### Sample size and power calculation

From correlation coefficient estimates obtained from the validation of the nutrients between questionnaires and biomarker concentrations, we estimated the sample size and power calculation from polyunsaturated fat. From the literature (Additional file 2: Table S1), the correlation coefficients of EPA and DHA are around 0.40. Applying the correlation coefficient as 0.4, we estimated that a sample size of 150 was likely to have enough power (90%) to test the significance level at 0.05.

## Results

Among the 306 participants sampled for this study, six had an estimated total energy intake outside the set allowable range (800–3245 kcal/day for men, 500–2842 kcal/day for women), and an additional three participants whose energy intake was beyond mean +/− 3 standard deviations were excluded from the analysis. Of the remaining 297 participants (47% women, average age 59.8 ± 12.8 years), all had complete FFQ data.

Table 1 shows the basic characteristics of the study participants, specified by gender. Compared with men, women were more likely to have a lower waist circumference, fasting glucose level, and uric acid level, yet higher triglycerides. In addition, the smoking and alcohol drinking rates were less for women than for men. The distributions of age, blood pressure, total cholesterol, HDL and LDL cholesterol, and medical history of hyperlipidemia, hypertension and type 2 diabetes were similar between genders, as were the percentages of lipid-lowering medications.

**Table 1:** General characteristics of the study participants by gender

	**Women (n = 140)**		**Men (n = 157)**		
	**Mean**	**SD**	**Mean**	**SD**	**P value**
Age (yr)	59.7	12.5	59.8	12.8	0.97
BMI (kg/m^2^)	19.9	2.7	20.5	2.5	0.08
Waist circumference (cm)	85.6	9.4	89.7	8.9	0.001
Systolic blood pressure (mmHg)	122.6	16.9	120.9	14.6	0.37
Diastolic blood pressure (mmHg)	73.6	11.0	75.1	12.2	0.25
Total cholesterol (mg/dL)	198.7	33.7	193.0	40.9	0.19
HDL cholesterol (mg/dL)	136.1	143.7	153.4	125.7	0.27
Triglyceride (mg/dL)	52.6	10.4	44.8	9.6	<.0001
LDL cholesterol (mg/dL)	120.9	31.1	119.6	34.2	0.73
Glucose (mg/dL)	98.0	14.2	102.0	17.0	0.031
Uric acid (mg/dL)	5.42	1.33	6.60	1.48	<.0001
Postprandial glucose (mg/dL)	134.5	53.6	153.2	96.4	0.18
HbA1c (%)	5.63	0.65	5.61	0.72	0.86
	%		%		
Current cigarette smoker	2.1		19.1		<.0001
Current drinking	2.9		31.2		<.0001
Hyperlipidemia	27.1		29.9		0.59
Hypertension	42.9		49.0		0.29
Type 2 diabetes	7.9		12.7		0.17
On Lipid-lowering medication					
Statins	21.4		22.3		0.86
Ezetimibe	2.1		3.2		0.58
Fibrates	1.4		4.5		0.13

BMI: body mass index; HbA1c: glycated hemoglobin; HDL: high density lipoprotein; LDL: low density lipoprotein; SD: standard deviation.

Table 2 shows various dietary components derived from the FFQs and plasma measurements of the study participants. Compared with men, women were more likely to have a lower total energy intake, and lower protein, fat, carbohydrate and cholesterol intake; however, the percentages of energy derived from protein and carbohydrates were similar between genders. In addition, the fish intake, including deep sea fish, was similar between genders. With regards to dietary fatty acid intake derived from the FFQ, women were more likely to eat less saturated fat than men. However, monounsaturated fat, polyunsaturated fat and EPA as well as DHA intakes were similar between genders.

**Table 2:** Dietary components derived from the FFQs and plasma measurements by gender

	**Women (n = 140)**		**Men (n = 157)**		
**FFQ**	**Mean**	**SD**	**Mean**	**SD**	**P value**
Total energy intake (kcal/d)	1306.7	420.8	1599.6	413.9	<.0001
Protein (g)	45.3	16.9	55.8	18.8	<.0001
Protein (% energy)	14.0	3.3	14.4	3.1	0.34
Fat (g)	46.6	19.2	51.8	19.2	0.020
Fat (% energy)	32.3	8.6	30.0	7.5	0.014
Carbohydrate (g)	176.0	65.1	216.4	67.1	<.0001
Carbohydrate (% energy)	53.6	10.3	55.6	9.4	0.09
Cholesterol (mg)	149.4	93.4	216.0	115.4	<.0001
Fish consumption serving (/d)					
Deep sea fish	0.49	0.76	0.51	0.74	0.82
Other fish	0.69	0.83	0.76	0.91	0.50
Seafood	0.15	0.27	0.25	0.46	0.034
Total fish & seafood	1.33	1.37	1.51	1.48	0.28
Saturated fat (g/d)	12.2	5.1	15.0	5.8	<.0001
Monounsaturated fat (g/d)	18.8	8.9	20.8	9.8	0.07
Polyunsaturated fat (g/d)	12.7	6.8	13.8	5.5	0.15
Saturated fat (% fat)	27.5	6.0	30.1	4.9	<.0001
Monounsaturated fat (% fat)	41.3	8.1	40.5	6.5	0.31
Polyunsaturated fat (% fat)	28.1	7.8	27.9	6.9	0.77
EPA (% fat)	0.48	0.46	0.46	0.40	0.77
C22:5 (% fat)	0.20	0.14	0.22	0.12	0.26
DHA (% fat)	0.77	0.65	0.78	0.54	0.89
Plasma concentration (/dL)					
Saturated fat (g)	1733	612	1774	606	0.56
Monounsaturated fat (g)	718	303	784	393	0.11
Polyunsaturated fat (g)	1726	487	1743	441	0.77
N-6 fatty acid	1511	442	1531	394	0.68
N-3 fatty acid	215.5	59.9	211.8	62.2	0.60
Marine fatty acid	147.9	45.4	146.9	47.2	0.85
EPA (20:5n-3)	20.7	6.4	19.4	4.0	0.034
DHA (22:6n-3)	127.2	42.3	127.5	46.0	0.95
Percentage of total fat, %					
Saturated fat	38.9	2.6	38.7	2.2	0.45
Monounsaturated fat	15.9	2.2	16.6	2.5	0.02
Polyunsaturated fat	39.2	3.5	38.8	3.8	0.29
N-6 fatty acid	34.3	3.4	34.1	3.6	0.56
N-3 fatty acid	4.93	0.89	4.72	0.86	0.04
Marine fatty acid	3.41	0.87	3.28	0.78	0.19
EPA (20:5n-3)	0.48	0.14	0.45	0.11	0.014
DHA (22:6n-3)	2.93	0.82	2.84	0.75	0.34

With regards to plasma fatty acid concentrations, women were more likely to have a higher percentage of fat intake from n-3 fatty acids, EPA and DHA, compared with men, although the absolute concentrations for both genders were similar. The correlation coefficients between dietary fishes and fatty acid intake as well as plasma fatty acid components in the study participants, after adjusting for gender, age, and total energy intake, are shown in Table 3. The coefficients between all kinds of fishes and marine n-3 fatty acid intake as well as plasma DHA ranged from 0.20 to 0.33 (*P* < 0.001). In addition, dietary EPA, C22:5 n-3 and DHA were correlated to plasma marine n-3 fatty acid and DHA, with partial Spearman correlation coefficients ranging from 0.26 to 0.35 (*P* < 0.001). In addition, the correlations from dietary fatty acids by either absolute intakes (g/day) and intakes as percentage of total energy intake were similar as the percentage of total fat intake (Additional file 2: Table S2 and S3).

**Table 3:** Spearman correlation coefficients between food-frequency questionnaire derived dietary fish as well as fatty acid compositions and plasma fatty acid compositions in all study participants, after adjusting for age, gender and total energy intake

**Questionnaire**	**Deep sea fish**	**Other fish**	**Seafood**	**Total fish & seafood**	**Saturated fat (% fat)**	**Monounsaturated fat (% fat)**	**Polyunsaturated fat (% fat)**	**C20:5 (% fat)**	**C22:5 (% fat)**	**C22:6 (% fat)**
**Plasma composition**										
Saturated fat	−0.125*	0.044	−0.053	−0.013	0.116*	−0.030	−0.007	−0.073	−0.055	−0.048
Monounsaturated fat	0.007	−0.042	0.003	−0.025	0.145*	0.054	−0.131*	0.001	0.060	0.019
Polyunsaturated fat	0.042	−0.004	0.036	0.012	−0.208***	−0.016	0.087	0.010	−0.040	−0.014
N-6 fatty acid	−0.002	−0.069	0.025	−0.056	−0.214***	−0.018	0.082	−0.067	−0.102	−0.090
N-3 fatty acid	0.193***	0.274***	0.035	0.313***	−0.016	0.043	0.032	0.334***	0.261***	0.321***
Marine fatty acid	0.196***	0.284***	0.038	0.318***	0.015	0.074	0.000	0.336***	0.271***	0.328***
EPA (20:5n-3)	−0.012	0.003	−0.040	0.023	−0.070	−0.076	0.091	−0.009	−0.034	−0.028
DHA (22:6n-3)	0.207***	0.294***	0.043	0.327***	0.021	0.088	−0.010	0.351***	0.287***	0.346***

The corresponding dietary intake includes all isomers of 20:1.

*: *P* < 0.05, **: *P* < 0.01, ***: *P* < 0.001.

With regards to the cross-classification of tabulations (Table 4) according to the total dietary fish and DHA intakes as well as plasma DHA quintiles, the diagonal proportions between fish intake and DHA ranged from 26% to 50%, and the highest kappa value was for total fish intake and DHA (0.63, 95% confidence interval, 0.58-0.68).

**Table 4:** Diagonal proportions in cross-classification of nutrient distribution and estimated kappa values and 95% confidence intervals for dietary intake of DHA, from fish intake, FFQs and plasma measurements in the study participants, categorized by quintile distribution

		**Diagonal proportion %**	**Kappa**	**SE**	**95% Conf limit**	
Total fish intake	C22:6	50.2	0.63	0.03	0.58	0.68
Total fish intake	DHA	26.9	0.18	0.04	0.10	0.26
C22:6	DHA	25.9	0.20	0.04	0.12	0.28

**Figure 1:**
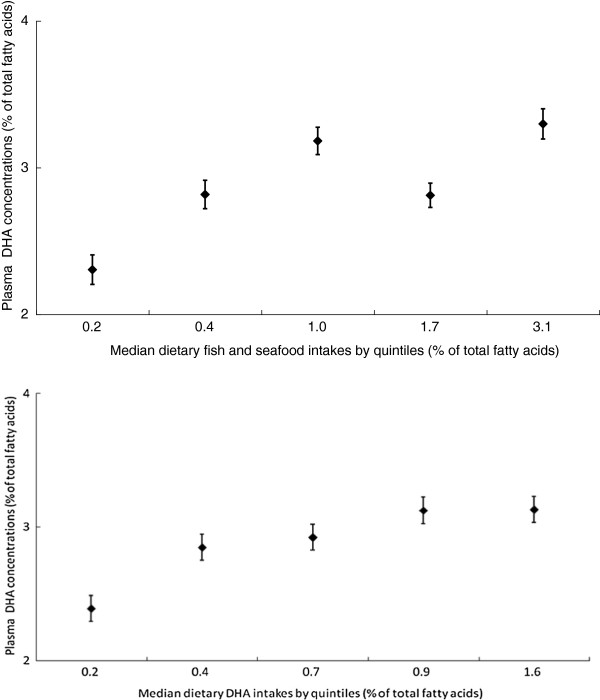
**Mean (± SEM) plasma DHA plotted against median daily fish and seafood (Upper) as well as DHA (Lower) intakes by quintile, after adjustment for age, sex, and total energy; both *P*** **< 0.001, test for trend.**

**Figure 2:**
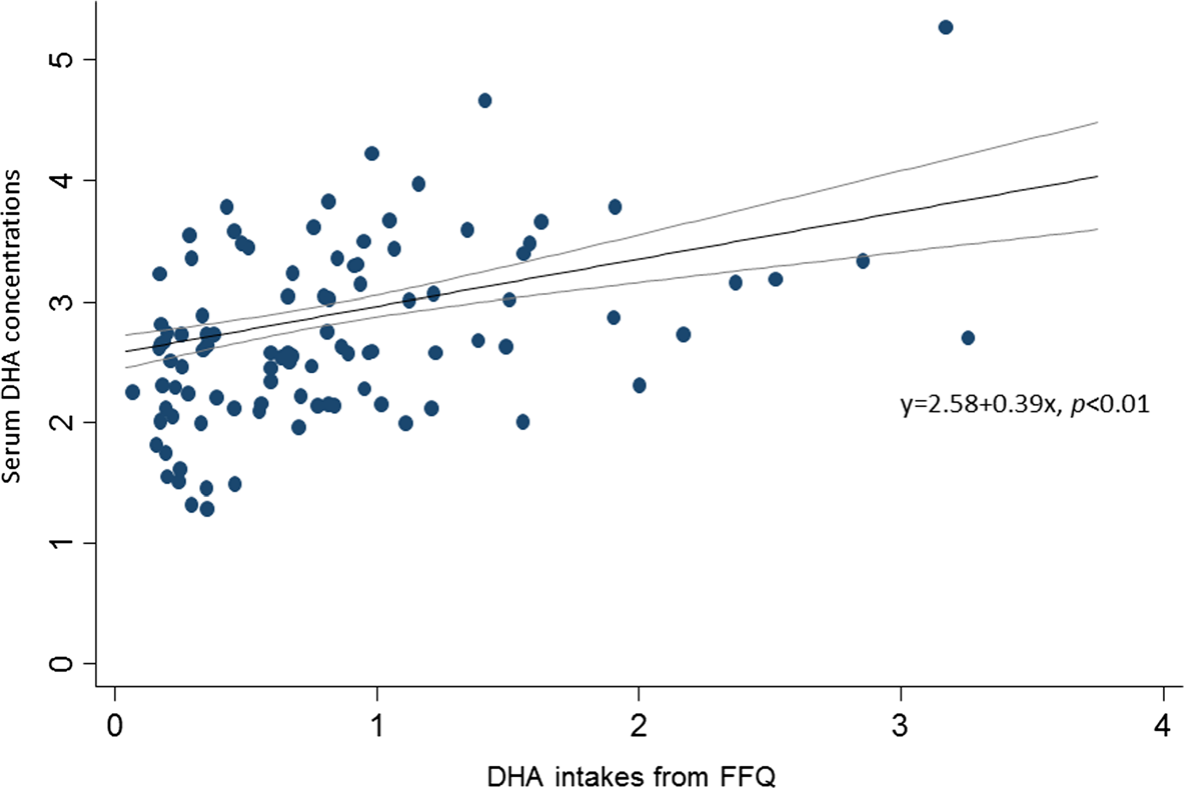
The Bland-Altman plot for the reliability of serum DHA concentration and intakes of DHA from FFQ and the estimated linear regression coefficients in the study participants.

The adjusted mean plasma DHA levels plotted against median daily fish as well as dietary DHA intakes by quintiles are shown in Figure 1. Compared with those in the lowest quintile, participants in the highest fish intake quintile had a significantly higher DHA level (adjusted mean difference, 0.99 ± 0.10%, test for trend, *p* < 0.001). We tested the non-linearity assumption of the quintiles and the linearity was not rejected, so that the dip in the fourth quintile for the relationship between dietary fish and seafood intake and DHA concentration may be due to a random error. Similar patterns between dietary DHA intake and plasma DHA level were found. However, the association between dietary fish intake and EPA was not significant (test for trend, *p* = 0.69). In addition, the Bland-Altman plot showed that the estimated from biomarker DHA concentrations were higher than the intakes from FFQ estimate: when the DHA intakes increased, the estimate of DHA concentration was higher than the intakes of DHA from FFQ (Figure 2).

## Discussion

In this cross-sectional study, we clearly demonstrated that dietary fish intakes measured from the interviewer-administered FFQ were correlated for plasma DHA levels among ethnic Chinese adults in Taiwan; however, the association for plasma EPA was not significant. In addition, a dose-response relationship between the FFQ and plasma DHA levels was shown.

The use of FFQs is feasible in epidemiological studies for the association between diet and disease. In a study by Arsenault *et al*., the correlation coefficients were 0.37 for EPA and 0.48 for DHA among 327 older adults (> = 65 yrs), and the magnitude was stable irrespective of the status of cognitive impairment [5]. Sullivan and colleagues conducted a validation study based on 53 healthy Australian adults to collect FFQs and 3-day weighed food records to estimate the long-chain n-3 polyunsaturated fatty acids and they found that the correlations were 0.62 for EPA and 0.72 for DHA [20]. In addition, this FFQ has been validated from plasma biomarker validation: the correlations were 0.54 for EPA and 0.48 for DHA [21]. Another validation study based on electronic FFQ, plasma biomarkers and weighted food records among 41 healthy adults showed high correlations for EPA and DHA [19].

Various resources have been used to provide the biochemical measurements, including adipose tissue, red blood cells, platelet membranes and subfractions of phospholipids (Additional file 2: Table S1). The correlation coefficients for EPA and DHA derived from adipose tissues have been found to be smaller than those for n-6 fatty acids and trans fats, and the correlation coefficients of n-3 fatty acids have been shown to range from 0.3 to 0.6. Only oil fish and EPA association was found among Australian population [15]. However, our study indicated that the coefficient was significant only for DHA, but not for EPA. From an European study, fish intake showed a statistically significant relationship with n-3 PUFA, EPA and DHA in serum [14]. These findings are consistent with previous literature based on middle American adults [4] and African Americans with prostate cancer [42]. Two possible explanations for the discrepancy between DHA and EPA coefficients are that firstly the proportion of DHA was much higher than that of EPA for the total fat contribution; and secondly that DHA was more biologically active than EPA due to its longer-chain characteristics [43]. Indeed, it was not clear why the proportion of DHA being higher would matter: the lower variability of EPA may better explain of a lack of association. And other sources of EPA that the questionnaire may have missed: Our data showed that dietary EPA values were less than other Asian populations.

The coefficients in our study were somewhat smaller than previous studies, especially for EPA. The validity of biochemical indicators is vulnerable to the problems of nutrient homeostatic mechanisms, bioavailability, time integration, medical condition, genetic backgrounds, and types of analytic procedures [11]. Admittedly, only a few biochemical indicators provide a sensitive and time-integrated reflection of nutrient intake. Our study indicated that plasma DHA, but not EPA, was related to dietary fish and marine n-3 fatty acid measurements. In addition, we did not consider the cod liver oil and n-3 fatty acid supplements because scanty data were available.

The association between DHA and EPA concentrations and lipid profiles in the general population is inconsistent. A population study based on Japanese and Americans showed that EPA was associated with HDL cholesterol only in Caucasians, but not in Japanese [6]. In addition, DHA was inversely associated with triglycerides in Caucasians, but not in Japanese. Our findings provided further evidence about the correlation of plasma fatty acid biomarkers as the surrogate indicators of dietary intakes [11], and contributed to the studies with the population with an Asian dietary habit. With regards to the reproducibility of the FFQ, our previous study [12] has shown that the FFQ is reproducible for Chinese-speaking people in Taiwan, and the correlation coefficients for n-3 fatty acids were similar to Sullivan and colleagues’ study [20].

This study has two strengths. First, we collected a well-established sample with archived clinical samples, adequate sample size, and extensive measures of various nutrient intakes and clinical information. Second, the participants were recruited from community and hospital settings, and the results can be applied to general practice. However, some limitations of this study should be mentioned. First, no other information, such as dietary record and recall, was available and the 32 items of questionnaire was relatively short form, so that the correlations were modest in strength although they were statistically significant. Second, only 3 questions for fish/seafood consumptions in the questionnaire may decrease the power of detecting dietary intake. Third, our study lacked the gold standard of the weighed food records data for intake of fatty acids. Finally, we did not measure the total energy expenditure, basal metabolic rate and energy intake among participants. Instead, we used the cutoff of convenient criteria from the Taiwanese community survey [12]. Goldberg and colleagues developed a feasible tool to assess the energy balance in populations [44] and evidence showed that the Goldberg cut-off for energy intake: basal metabolic rate information was a good indicator to define the under-, acceptable- and over-reporters for diet intake [45].

## Conclusion

In conclusion, our study demonstrated that the dietary intakes of fish and of long chain n-3 fatty acids, as determined by our food frequency questionnaire, are correlated with the percentages of these fatty acids in plasma, and in particular with plasma DHA.

## Abbreviations

DHA: Docosahexaenoic acid; EPA: Eicosapentaenoic acid; FFQ: Food frequency questionnaire.

## Competing interests

The authors declare no competing interests.

## Authors’ contributions

KLC carried out the study design, data collection and analysis, and wrote the draft. MSL provided the food frequency questionnaire and test the correlation. PRC provided the nutritional survey and revised draft. HCH carried out the laboratory measurements and quality control and assurance. YTL participated in the design of the study and revised the draft. MFC conceived of the study, and participated in its design and coordination and helped to draft the manuscript. All authors read and approved the final manuscript.

## Funding

This work was supported in part by a grant of National Science Council, Taiwan (NSC 97-2314-B-002 -130 -MY3, NSC 98-2911-I-002-062) and National Taiwan University Hospital, Taiwan (98-S1056).

## Pre-publication history

The pre-publication history for this paper can be accessed here:

http://www.biomedcentral.com/1471-2288/13/23/prepub

## Supplementary Material

Additional file 1 A translation of the Chinese version of the 32-item FFQ. Click here for file

Additional file 2: Table S1 Literature review comparing measures of dietary fatty acid intake by biochemical indicators, food frequency questionnaire (FFQ) and diet record (DR) methods. **Table S2:** Spearman correlation coefficients between the dietary fish and fatty acids by absolute intake (g/day) food-frequency questionnaire and plasma fatty acid components (g) in the study participants, after adjusting for age, gender and total energy intake. **Table S3: **Spearman correlation coefficients between the dietary fish and fatty acids by the percentage of total energy intake in the food-frequency questionnaire and plasma fatty acid concentrations in the study participants, after adjusting for age and gender. Click here for file

## References

[B1] Tavazzi L Maggioni AP Marchioli R Barlera S Franzosi MG Latini R Lucci D Nicolosi GL Porcu M Tognoni G Gissi-HF Investigators Effect of n-3 polyunsaturated fatty acids in patients with chronic heart failure (the GISSI-HF trial): a randomised, double-blind, placebo-controlled trial Lancet 2008 372 9645 1223 1230 10.1016/S0140-6736(08)61239-8 18757090

[B2] Baylin A Kabagambe EK Siles X Campos H Adipose tissue biomarkers of fatty acid intake Am J Clin Nutr 2002 76 4 750 757 12324287 10.1093/ajcn/76.4.750

[B3] Baylin A Kim MK Donovan-Palmer A Siles X Dougherty L Tocco P Campos H Fasting whole blood as a biomarker of essential fatty acid intake in epidemiologic studies: comparison with adipose tissue and plasma Am J Epidemiol 2005 162 4 373 381 10.1093/aje/kwi213 16014782

[B4] Ma J Folsom AR Shahar E Eckfeldt JH Plasma fatty acid composition as an indicator of habitual dietary fat intake in middle-aged adults. The atherosclerosis risk in communities (ARIC) study investigators Am J Clin Nutr 1995 62 3 564 571 7661118 10.1093/ajcn/62.3.564

[B5] Arsenault LN Matthan N Scott TM Dallal G Lichtenstein AH Folstein MF Rosenberg I Tucker KL Validity of estimated dietary eicosapentaenoic acid and docosahexaenoic acid intakes determined by interviewer-administered food frequency questionnaire among older adults with mild-to-moderate cognitive impairment or dementia Am J Epidemiol 2009 170 1 95 103 10.1093/aje/kwp089 19433614 PMC2733037

[B6] Motoyama KR Curb JD Kadowaki T El-Saed A Abbott RD Okamura T Evans RW Nakamura Y Sutton-Tyrrell K Rodriquez BL Association of serum n-6 and n-3 polyunsaturated fatty acids with lipids in 3 populations of middle-aged men Am J Clin Nutr 2009 90 1 49 55 10.3945/ajcn.2008.26761 19474136 PMC2696994

[B7] Kaaks R Riboli E Validation and calibration of dietary intake measurements in the EPIC project: methodological considerations. European prospective investigation into cancer and nutrition Int J Epidemiol 1997 26 Suppl 1 S15 S25 9126530 10.1093/ije/26.suppl_1.s15

[B8] Kaaks RJ Biochemical markers as additional measurements in studies of the accuracy of dietary questionnaire measurements: conceptual issues Am J Clin Nutr 1997 65 4 Suppl 1232S 1239S 9094927 10.1093/ajcn/65.4.1232S

[B9] Hodson L Skeaff CM Fielding BA Fatty acid composition of adipose tissue and blood in humans and its use as a biomarker of dietary intake Prog Lipid Res 2008 47 5 348 380 10.1016/j.plipres.2008.03.003 18435934

[B10] Sun Q Ma J Campos H Hankinson SE Hu FB Comparison between plasma and erythrocyte fatty acid content as biomarkers of fatty acid intake in US women Am J Clin Nutr 2007 86 1 74 81 17616765 10.1093/ajcn/86.1.74

[B11] Hunter D Willett WC Biochemical indicators of dietary intake Nutritional epidemiology 1998 2 New York: Oxford University Press 174 243

[B12] Lee MS Pan WH Liu KL Yu MS Reproducibility and validity of a Chinese food frequency questionnaire used in Taiwan Asia Pac J Clin Nutr 2006 15 2 161 169 16672199

[B13] Hjartaker A Lund E Bjerve KS Serum phospholipid fatty acid composition and habitual intake of marine foods registered by a semi-quantitative food frequency questionnaire Eur J Clin Nutr 1997 51 11 736 742 10.1038/sj.ejcn.1600475 9368807

[B14] Amiano P Dorronsoro M de Renobales M de Gordoa JC R Irigoien I Very-long-chain omega-3 fatty acids as markers for habitual fish intake in a population consuming mainly lean fish: the EPIC cohort of gipuzkoa. European prospective investigation into cancer and nutrition Eur J Clin Nutr 2001 55 10 827 832 10.1038/sj.ejcn.1601242 11593343

[B15] Mina K Fritschi L Knuiman M A valid semiquantitative food frequency questionnaire to measure fish consumption Eur J Clin Nutr 2007 61 8 1023 1031 10.1038/sj.ejcn.1602617 17299496

[B16] Fuhrman BJ Barba M Krogh V Micheli A Pala V Lauria R Chajes V Riboli E Sieri S Berrino F Erythrocyte membrane phospholipid composition as a biomarker of dietary fat Ann Nutr Metab 2006 50 2 95 102 10.1159/000090496 16373991

[B17] Garaulet M Perez-Llamas F Perez-Ayala M Martinez P de Medina FS Tebar FJ Zamora S Site-specific differences in the fatty acid composition of abdominal adipose tissue in an obese population from a Mediterranean area: relation with dietary fatty acids, plasma lipid profile, serum insulin, and central obesity Am J Clin Nutr 2001 74 5 585 591 11684525 10.1093/ajcn/74.5.585

[B18] Garland M Sacks FM Colditz GA Rimm EB Sampson LA Willett WC Hunter DJ The relation between dietary intake and adipose tissue composition of selected fatty acids in US women Am J Clin Nutr 1998 67 1 25 30 9440371 10.1093/ajcn/67.1.25

[B19] Swierk M Williams PG Wilcox J Russell KG Meyer BJ Validation of an Australian electronic food frequency questionnaire to measure polyunsaturated fatty acid intake Nutrition 2011 27 6 641 646 10.1016/j.nut.2010.06.011 20869207

[B20] Sullivan BL Brown J Williams PG Meyer BJ Dietary validation of a new Australian food-frequency questionnaire that estimates long-chain n-3 polyunsaturated fatty acids Br J Nutr 2008 99 3 660 666 17903342 10.1017/S0007114507837408

[B21] Sullivan BL Williams PG Meyer BJ Biomarker validation of a long-chain omega-3 polyunsaturated fatty acid food frequency questionnaire Lipids 2006 41 9 845 850 10.1007/s11745-006-5039-0 17152921

[B22] Chang SC Lee MS Li CH Chen ML Dietary fiber content and composition of vegetables in Taiwan area Asia Pac J Clin Nutr 1995 4 204 210 24393673

[B23] Chang SC Lee MS Lin CJ Chen ML Dietary fiber contents and composition of fruits in Taiwan area Asia Pac J Clin Nutr 1998 7 206 210 24393673

[B24] Lyu LC Huang SH Hsu CY Lee MS Lin SH Inter-relationships of nutrient intakes for urban Chinese spouses in Taiwan Int J Food Sci Nutr 2004 55 3 227 236 10.1080/09637480410001733851 15223600

[B25] Chien KL Chen MF Hsu HC Chang WT Su TC Lee YT Hu FB Plasma uric acid and the risk of type 2 diabetes in a chinese community Clin Chem 2008 54 2 310 316 10.1373/clinchem.2007.095190 18089655

[B26] Chien KL Sung FC Hsu HC Su TC Lin RS Lee YT Apolipoprotein A1 & B, and stroke events in a community-based cohort in taiwan: report of chin-shan community cardiovascular study Stroke 2002 33 39 44 10.1161/hs0102.101626 11779886

[B27] Lee YT Lin RS Sung FC Yang CY Chien KL Chen WJ Su TC Hsu HC Huang YC Chin-shan community cardiovascular cohort in Taiwan: baseline data and five-year follow-up morbidity and mortality J Clin Epidemiol 2000 53 836 846 10.1016/s0895-4356(00)00198-0 10942867

[B28] Chen PR Chien KL Su TC Chang CJ Liu TL Cheng HC Tsai HC Dietary sesame reduces serum cholesterol and enhances antioxidant capacity in hypercholesterolemia Nutr Res 2005 25 6 559 567 10.1016/j.nutres.2005.05.007

[B29] Chien KL Hsu HC Sung FC Su TC Chen MF Lee YT Hyperuricemia as a risk factor on cardiovascular events in Taiwan: the chin-shan community cardiovascular cohort study Atherosclerosis 2005 183 147 155 10.1016/j.atherosclerosis.2005.01.018 16154134

[B30] Lee MM Pan WH Yu SL Huang PC Foods predictive of nutrient intake in Chinese diet in Taiwan: I. Total calories, protein, fat and fatty acids Int J Epidemiol 1992 21 5 922 928 10.1093/ije/21.5.922 1468854

[B31] Pan WH Lee MM Yu SL Huang PC Foods predictive of nutrient intake in chinese diet in Taiwan: II. Vitamin a, vitamin B1, vitamin B2, vitamin C and calcium Int J Epidemiol 1992 21 5 929 934 10.1093/ije/21.5.929 1468855

[B32] Department of Health Nutrient composition data bank for foods in Taiwan area (in Chinese) 1998 Taipei: Department of Health, Republic of China

[B33] Huang PC Wei HN Huang SC Yu SL Composition of foods used in Taiwan-supplements (in chinese) J Chin Nutr Soc 1978 3 11 15

[B34] Tung TC Huang PC Li HC Chen HL Composition of foods used in Taiwan (in Chinese) J Formosa Med Assoc 1961 60 973 1005 13923088

[B35] Chinese Academy of Preventive Medicine IoNaFH Food composition table (in Chinese) 1991 Beijing: People’s Health Press

[B36] Lopes-Virella M Stone P Ellis S Colwell JA Cholesterol determination in high-density lipoproteins separated by three different methods Clin Chem 1977 23 5 882 884 192488

[B37] Friedewald WT Levy RI Fredrickson DS Estimation of the concentration of low-density lipoprotein cholesterol in plasma, without use of the preparative ultracentrifuge Clin Chem 1972 18 6 499 502 4337382

[B38] Hunter DJ Rimm EB Sacks FM Stampfer MJ Colditz GA Litin LB Willett WC Comparison of measures of fatty acid intake by subcutaneous fat aspirate, food frequency questionnaire, and diet records in a free-living population of US men Am J Epidemiol 1992 135 4 418 427 1550093 10.1093/oxfordjournals.aje.a116302

[B39] Willett W Stampfer MJ Total energy intake: implications for epidemiologic analyses Am J Epidemiol 1986 124 1 17 27 3521261 10.1093/oxfordjournals.aje.a114366

[B40] Fleiss JL The design and analysis of clinical experiments 1986 New York: John Wiley & Sons

[B41] Signorello LB Buchowski MS Cai Q Munro HM Hargreaves MK Blot WJ Biochemical validation of food frequency questionnaire-estimated carotenoid, {alpha}-tocopherol, and folate intakes among african americans and non-hispanic whites in the southern community cohort study Am J Epidemiol 2010 171 4 488 497 10.1093/aje/kwp402 20061366 PMC2842194

[B42] Holmes MD Powell IJ Campos H Stampfer MJ Giovannucci EL Willett WC Validation of a food frequency questionnaire measurement of selected nutrients using biological markers in African-American men Eur J Clin Nutr 2007 61 11 1328 1336 10.1038/sj.ejcn.1602641 17299490

[B43] Khairallah RJ O’Shea KM Brown BM Khanna N Des Rosiers C Stanley W Treatment with docosahexaenoic acid, but Not eicosapentaenoic acid, delays Ca2 + −induced mitochondria permeability transition in normal and hypertrophied myocardium J Pharmacol Exp Ther 2010 335 1 155 162 10.1124/jpet.110.170605 20624993 PMC2957778

[B44] Goldberg GR Prentice AM Coward WA Davies HL Murgatroyd PR Sawyer MB Ashford J Black AE Longitudinal assessment of the components of energy balance in well-nourished lactating women Am J Clin Nutr 1991 54 5 788 798 1951148 10.1093/ajcn/54.5.788

[B45] Black AE The sensitivity and specificity of the Goldberg cut-off for EI:BMR for identifying diet reports of poor validity Eur J Clin Nutr 2000 54 5 395 404 10.1038/sj.ejcn.1600971 10822286

