# Supplementary material for: A Taiwanese food frequency questionnaire correlates with plasma docosahexaenoic acid but not with plasma eicosapentaenoic acid levels: questionnaires and plasma biomarkers
Source: BMC Med Res Methodol. 2013 Feb 16;13:23. doi: 10.1186/1471-2288-13-23 (PMC3598308; doi:10.1186/1471-2288-13-23)
Supplement: Additional file 1 — A translation of the Chinese version of the 32-item FFQ. [file 1471-2288-13-23-S1.doc]

**Additional file 1:**

**Supplementary Materials: A translation of the Chinese version of the 32-item FFQ**

I would like to ask you about your dietary habits over the last year. Please answer the following questions as best you can.

1. Are you a total vegetarian? If “Yes”, how long have you been a vegetarian?
2. Regarding milk, including cows milk, goats milk and yogurt milk. If we consider 240 cc as one cup of milk or yogurt milk, and four spoons of canned dried milk as one cup, on average, how much milk per week did you consume over the last year?
3. Regarding dairy products, including yogurt and cheese. If we consider 100 gm of yogurt or one piece of cheese to be one serving, on average, how much dairy product per week did you consume in the last year?
4. Regarding eggs, including chicken eggs, preserved eggs and goose eggs. If we consider one egg or five smaller bird eggs as one serving, on average, how much egg per day did you consume in the last year?
5. Regarding livestock meat, i.e., 4-legged animals, including pork, beef and mutton. If we consider two slices of meat (as on the indicator card), four pieces of meat, or palm-sized spare ribs (bone-carrying meat) as one serving, on average, how much did you consume in the last year?
6. Regarding poultry, i.e., 2-legged animals, including chicken, duck and goose. Please look at the indicator card. If we consider one leg, one breast, or half-sized fried chicken ribs as one serving, on average, how much per week did you consume per week in the last year?
7. Regarding deep sea fish, such as codfish, herring, tuna and salmon. If we consider two herring or two pieces of tuna as one serving, on average, how much deep sea fish per week did you consume in the last year?
8. Regarding other fish besides deep sea fish, including those from farms and fresh water fish, such as mouthbreeder, hair-tail or mackerel pike (samba fish). If we consider one piece of mouthbreeder or hair-tail fish, or one mackerel pike fish as one serving, on average, how much per week did you consume in the last year?
9. Regarding other seafood such as shrimps, oysters, clams or cuttlefish. If we consider 4 shrimps, 20 oysters, 6 clams or two 12-cm long pieces of cuttlefish as one serving, on average, how much per week did you consume in the last year?
10. Regarding animal livers, including chicken, pork and beef livers. If we consider half a cup as one serving, on average, how much per week did you consume in the last year?
11. Regarding other animal organs, including pork stomach, beef stomach, intestines, hearts, kidneys or chicken hearts. If we consider two pieces of stomach (as on the indicator card) or eight chicken hearts as one serving, on average, how much per week did you consume in the last year?
12. Regarding soybean milk, if we consider a 240 cc cup as one serving, on average, how much soybean milk per week did you consume in the last year?
13. Regarding soybean products, including tender tofu, five-spice dried tofu or traditional tofu. If we consider half a box of tender tofu, two pieces of five-spice tofu or one-cm thickness traditional bean curd as one serving, on average, how much did you consume in the last year?
14. Regarding light-colored vegetables, including cabbage, bok choy, cauliflower, chervil, squashes and mushrooms. If we consider a half cup of these vegetables as one serving, on average, how much did you consume in the last year?
15. Regarding spinach and broccoli. If we consider half a cup of these vegetables as one serving, on average, how much did you consume in the last year?
16. Regarding other deep-colored vegetables apart from spinach and broccoli, including Chinese broccoli, lettuce, water convolvulus, green pepper, aubergine or bugleweed. If we consider half a cup of these vegetables as one serving, on average, how much per day did you consume in the last year?
17. Regarding carrots and pumpkin. If we consider half a cup of these vegetables as one serving, on average, how much per day did you consume in the last year?
18. Do you usually cook vegetables in oil? When cooked, do you add additional oils, marinated ground pork or mayonnaise?
19. Regarding tomatoes. If we consider one large tomato, 15 cherry tomatoes or half a cup (120 cc) of tomato juice as one serving, on average, how much per week did you consume in the last year?
20. Regarding citrus fruits in the productive seasons, such as tangerine, orange, grapefruit. If we consider one segment of tangerine or orange, half a segment of a big grapefruit, or half a cup (120 cc) fruit juice as one serving, on average, how much per week did you consume in the last year?
21. Regarding other fruits including persimmons, papayas, and mangoes in the productive seasons. If we consider one persimmon, a quarter of a middle-sized papaya or half a mango as one serving, on average, how much per week did you consume in the last year?
22. Do you eat fruit everyday? If not, how often do you eat fruit? If we consider half a guava, one apple or kiwi fruit, or 12 grapes or longans as one serving, on average, how much per day did you consume in the last year?
23. Regarding rice and wheat products. If we consider one cup of rice, two cups of noodles, two pieces of bread, or one steamed bun as one serving, on average, how much per day did you consume in the last year?
24. Regarding other types of grain, such as brown rice, acrospires, or whole wheat, on average, how much per day did you consume in the last year?
25. Do you frequently eat fried rice, fried noodles, or oil-added rice or noodles?
26. Do you eat instant noodles? (If yes.) If we consider one pack of instant noodles as one serving, on average, how much per week did you consume in the last year?
27. Regarding root and stem vegetables, including ipomoea, potatoes, taro, water caltrop and corn. If we consider one cup of these vegetables as one serving, on average, how much per day did you consume in the last year?
28. Regarding bread and cakes, such as crisp milk bread or taro bake. If we consider one piece of bread or cake as one serving, on average, how much per day did you consume in the last year?
29. Regarding sugar-containing beverages, such as coffee, coco, and tea with added sugar, fruit juice, adzuki bean tea, mung bean tea, and store-bought beverages. If we consider one 240 cc cup as one serving, on average, how much per week did you consume in the last year?
30. Regarding fried or deep-fried foods, including chicken steak, pork steak or fried fish. If we consider half a dish as one serving, on average, how much per week did you consume in the last year?
31. Where do you eat most frequently, at home or outside? How often do you eat at home compared to going out (a percentage)? Which cooking oil do you use most frequently? Which oil do you use most frequently after this? What are the proportions of use of these two cooking oils?
32. Do you take dietary nutrient replacements, including Centro(a Taiwanese brand), Vitamin A, C or E, gingko, evening primrose oil, or glucosamine (Viartril-s)? If you have taken any, on average, how much per week did you consume in the last year?
