# Supplementary material for: A Taiwanese food frequency questionnaire correlates with plasma docosahexaenoic acid but not with plasma eicosapentaenoic acid levels: questionnaires and plasma biomarkers
Source: BMC Med Res Methodol. 2013 Feb 16;13:23. doi: 10.1186/1471-2288-13-23 (PMC3598308; doi:10.1186/1471-2288-13-23)
Supplement: Additional file 2: Table S1 — Literature review comparing measures of dietary fatty acid intake by biochemical indicators, food frequency questionnaire (FFQ) and diet record (DR) methods. Table S2: Spearman correlation coefficients between the dietary fish and fatty acids by absolute intake (g/day) food-frequency questionnaire and plasma fatty acid components (g) in the study participants, after adjusting for age, gender and total energy intake. Table S3: Spearman correlation coefficients between the dietary fish and fatty acids by the percentage of total energy intake in the food-frequency questionnaire and plasma fatty acid concentrations in the study participants, after adjusting for age and gender. [file 1471-2288-13-23-S2.doc]

**Additional File 2, Table S**1: Literature review comparing measures of dietary fatty acid intake by biochemical indicators, food frequency questionnaire (FFQ) and diet record (DR) methods

| **Author, year** | **Study participants characteristics** | **Biochemical sample origin** | **FFQ, DR methods** | **Statistics** | **Results** | **Comments** |
| --- | --- | --- | --- | --- | --- | --- |
| London et al. 1991 | USA, postmenopausal women, n=115 | Adipose tissue | FFQ: 116 items |  | EPA, DHA, r=0.40 |  |
| Hunter et al. 1992 | USA, Boston-area men aged 40-75 years in the HPFS, n=118 | Fat aspirate from the lateral buttock | FFQ: 131 items  DR: two specified 1-week periods, 7 months apart | 1. Spearman correlation coefficient  2. Cross-classified quintiles | With DR: Saturated fat, r=0.16  Monounsaturated fat, r=0.22  Polyunsaturated fat, r=0.49  With FFQ: Saturated fat, r=.18  Monounsaturated fat, r=0.14  Polyunsaturated fat, r=0.50  EPA: r=0.47 | Underestimate EPA: % of total fat, 0.19-0.21 in FFQ vs. 0.06 in fat aspirate, similar estimate of polyunsaturated fat, 22-23% |
| Ma et al. 1995 | USA, 3570 middle-aged adults from the ARIC study | Plasma phospholipids and cholesterol ester (CE),  Gas chromatography | FFQ: 66-item | Pearson coefficient, Correction by test-retest reliability coefficient | For phospholipids and CE: saturated (0.15, 0.23), monounsaturated (0.05, 0.01), polyunsaturated (0.25, 0.31), linoleic acid (0.22, 0.28), linolenic acid (0.15, 0.21), EPA (0.20, 0.23), DHA (0.42, 0.42) | Underestimate of EPA & DHA by FFQ |
| Marckman et al. 1995 | USA, 27 adults, 20-29 years | *Erythrocyte phospholipids* between 8 months, adipose tissue | Three 7-day weighted food records |  | Biochemical indicator 8-m separately : EPA: 0.68, DHA: 0.93  Adipose vs. food records: EPA, 0.40, DHA:L 0.66 |  |
| Prisco et al. 1996 | 20 healthy men | Plasma and erythrocyte phospholipids | 4 months after supplemental capsules, 3 months wash-out | Change of concentration before and after supplements |  | Interventional trial |
| Garland et al. 1998 | 140 nurses in Health Nurse Study | Fat aspirate collection | 2 dietary records, 2 FFQ | Spearman correlation coefficient | 0.40 for PUFA, not specified for DHA and EPA |  |
| Baylin et al. 2002 | 503 Costa Rican adults (136 women), mean 56 yrs for men and 60 yrs for women | Adipose tissue | 135-item FFQ | Partial Spearman correlation | 0.15 for EPA, 0.18 for DHA  Saturated fat: 0.18  N-6 fatty acids: 0.24-0.58  Trans fat: 0.16-0.58 | Small values for n-3 fatty acids than n-6 fatty acids and trans fat |
| Ritter-Gooder et al. 2006 | USA, Midwest cardiac patients, women: men=17:11, 43-77 years | Not available | 24-hour food recall  FFQ: 152 items | Pearson correlation  Cochrane coefficient, α | n-3 fatty acids, r=0.42  α=0.83 | Validity of FFQ among specific cardiac patients |
| Holmes et al. 2007 | 104 African Americans with prostate cancer, | Adipose tissue | FFQ: 158 items | Spearman correlation coefficient, | 0.19 for EPA (not significant)  0.47 for DHA (significant) | Plasma for lycopene, beta carotene and alpha tocopherol intake; adipose for fatty acid |
| Arsenault et al. | 273 community-dwelling adults, aged >=60 yrs | Plasma | FFQ: 4 questions for fish and seafood consumption | Age- and energy-adjusted Pearson correlation  Quartile classification | 0.37 for EPA  0.48 for DHA | Stratified by cognition status  Cognitive function and clinical diagnosis of dementia did not affect the correlations |
| This study, 2011 | Taiwan, 300 participants, 35-75 years | Plasma | FFQ: 38 items | Partial Spearman correlation, kappa, quintile classification, regression model | 0.02 for EPA (not significant)  0.33 for DHA (significant) | General clinical setting |

Abbreviations: ARIC: Atherosclerosis Risk in Community; DHA: docosahexaenoic acid, DR: diet record; EPA: eicosapentaenoic acid; FFQ: food frequency questionnaire; HPFS: Health Professional Follow-up Study;

**Additional File 2, Table S2: Spearman correlation coefficients between the dietary fish and fatty acids by absolute intake (g/day) food-frequency questionnaire and plasma fatty acid components (g) in the study participants, after adjusting for age, gender and total energy intake**

|  | Deep sea fish (g) | | Other fish (g) | | Seafood | (g) | Total fish & seafood (g) | Saturated fat (g) | | Monounsaturated fat (g) | | Polyunsaturated fat (g) | | | C20:5 (mg) | | | C22:5 (mg) | | | | C22:6 (mg) | | |
| --- | --- | --- | --- | --- | --- | --- | --- | --- | --- | --- | --- | --- | --- | --- | --- | --- | --- | --- | --- | --- | --- | --- | --- | --- |
| Saturated fat (g) | 0.007 |  | 0.023 |  | -0.017 |  | 0.030 |  | -0.020 |  | 0.001 |  | -0.080 |  | | 0.028 |  | | 0.050 |  | 0.042 | |  |  |
| Monounsaturated fat (g) | 0.027 |  | -0.025 |  | 0.012 |  | 0.001 |  | 0.009 |  | 0.051 |  | -0.097 |  | | 0.021 |  | | 0.056 |  | 0.033 | |  |  |
| Polyunsaturated fat (g) | 0.042 |  | 0.008 |  | 0.009 |  | 0.021 |  | -0.053 |  | 0.036 |  | -0.036 |  | | 0.038 |  | | 0.057 |  | 0.043 | |  |  |
| N-6 fattt acid (g) | 0.022 |  | -0.030 |  | 0.002 |  | -0.019 |  | -0.044 |  | 0.044 |  | -0.020 |  | | 0.000 |  | | 0.028 |  | 0.005 | |  |  |
| N-3 fatty acid (g) | 0.157 | ** | 0.214 | *** | 0.012 |  | 0.238 | *** | -0.053 |  | -0.017 |  | -0.101 |  | | 0.247 | *** | | 0.215 | *** | 0.251 | | *** |  |
| Marine fatty acid (g) | 0.192 | *** | 0.258 | *** | 0.015 |  | 0.282 | *** | -0.035 |  | 0.006 |  | -0.110 |  | | 0.294 | *** | | 0.257 | *** | 0.301 | | *** |  |
| EPA (20:5n-3) (g) | 0.041 |  | 0.009 |  | -0.025 |  | 0.046 |  | 0.003 |  | 0.044 |  | 0.059 |  | | 0.052 |  | | 0.097 |  | 0.058 | |  |  |
| DHA (22:6n-3) (g) | 0.195 | *** | 0.257 | *** | 0.024 |  | 0.283 | *** | -0.038 |  | 0.003 |  | -0.117 | * | | 0.295 | *** | | 0.253 | *** | 0.301 | | *** |  |

*: *P*<0.05, **: *P*<0.01, ***: *P*<0.001

**Additional File 2, Table S3: Spearman correlation coefficients between the dietary fish and fatty acids by the percentage of total energy intake in the food-frequency questionnaire** and plasma fatty acid concentrations in the study participants, after adjusting for age and gender.

|  | Deep sea fish (/total energy) | | Other fish (/total energy) | | Seafood (/total energy) | | Total fish & seafood (/total energy) | | Saturated fat (/total energy) | | Monounsaturated fat (/total energy) | | Polyunsaturated fat (/total energy) | | C20:5 (/total energy) | | C22:5 (/total energy) | | C22:6 (/total energy) | |
| --- | --- | --- | --- | --- | --- | --- | --- | --- | --- | --- | --- | --- | --- | --- | --- | --- | --- | --- | --- | --- |
| Saturated fat (g) | -0.002 |  | 0.028 |  | -0.017 |  | 0.018 |  | -0.028 |  | -0.013 |  | -0.067 |  | 0.020 |  | 0.035 |  | 0.030 |  |
| Monounsaturated fat (g) | 0.024 |  | -0.023 |  | 0.014 |  | -0.013 |  | -0.002 |  | 0.035 |  | -0.078 |  | 0.011 |  | 0.043 |  | 0.019 |  |
| Polyunsaturated fat (g) | 0.040 |  | 0.011 |  | 0.007 |  | 0.010 |  | -0.064 |  | 0.024 |  | -0.016 |  | 0.038 |  | 0.048 |  | 0.038 |  |
| N-6 fattt acid (g) | 0.019 |  | -0.028 |  | 0.000 |  | -0.032 |  | -0.060 |  | 0.030 |  | -0.002 |  | -0.001 |  | 0.016 |  | -0.002 |  |
| N-3 fatty acid (g) | 0.165 | ** | 0.223 | *** | 0.015 |  | 0.247 | *** | -0.038 |  | -0.018 |  | -0.072 |  | 0.258 | *** | 0.236 | *** | 0.259 | *** |
| Marine fatty acid (g) | 0.198 | *** | 0.264 | *** | 0.015 |  | 0.293 | *** | -0.015 |  | 0.003 |  | -0.082 |  | 0.306 | *** | 0.282 | *** | 0.309 | *** |
| EPA (20:5n-3) | 0.046 |  | 0.018 |  | -0.034 |  | 0.045 |  | 0.017 |  | 0.029 |  | 0.044 |  | 0.051 |  | 0.086 |  | 0.048 |  |
| DHA (22:6n-3) | 0.200 | *** | 0.263 | *** | 0.023 |  | 0.295 | *** | -0.019 |  | 0.000 |  | -0.087 |  | 0.307 | *** | 0.279 | *** | 0.310 | *** |
| Saturated fat (% of total fat) | -0.135 | * | 0.042 |  | -0.065 |  | -0.023 |  | 0.023 |  | -0.061 |  | -0.070 |  | -0.086 |  | -0.060 |  | -0.062 |  |
| Monounsaturated fat (% of total fat) | 0.020 |  | -0.036 |  | 0.024 |  | -0.030 |  | 0.023 |  | 0.018 |  | -0.099 |  | -0.008 |  | 0.031 |  | 0.005 |  |
| Polyunsaturated fat (% of total fat) | 0.046 |  | -0.004 |  | 0.031 |  | 0.023 |  | -0.024 |  | 0.047 |  | 0.132 | * | 0.040 |  | 0.006 |  | 0.022 |  |
| N-6 fatty acid (% of total fat) | -0.001 |  | -0.070 |  | 0.021 |  | -0.052 |  | -0.014 |  | 0.065 |  | 0.148 | * | -0.033 |  | -0.052 |  | -0.048 |  |
| N-3 fatty acid (% of total fat) | 0.209 | *** | 0.281 | *** | 0.038 |  | 0.343 | *** | 0.001 |  | 0.000 |  | 0.007 |  | 0.328 | *** | 0.267 | *** | 0.321 | *** |
| Marine fatty acid (% of total fat) | 0.207 | *** | 0.288 | *** | 0.033 |  | 0.342 | *** | 0.018 |  | 0.016 |  | -0.018 |  | 0.329 | *** | 0.268 | *** | 0.324 | *** |
| EPA (20:5n-3) (% of total fat) | 0.000 |  | 0.007 |  | -0.044 |  | 0.035 |  | 0.027 |  | -0.005 |  | 0.080 |  | 0.016 |  | 0.021 |  | 0.010 |  |
| DHA (22:6n-3) (% of total fat) | 0.216 | *** | 0.298 | *** | 0.038 |  | 0.352 | *** | 0.011 |  | 0.018 |  | -0.030 |  | 0.340 | *** | 0.277 | *** | 0.336 | *** |

*: *P*<0.05, **: *P*<0.01, ***: *P*<0.001

**Reference:**

1. London SJ, Sacks FM, Caesar J, Stampfer MJ, Siguel E, Willett WC: **Fatty acid composition of subcutaneous adipose tissue and diet in postmenopausal US women**. *Am J Clin Nutr* 1991, **54**(2):340-345.

2. Hunter DJ, Rimm EB, Sacks FM, Stampfer MJ, Colditz GA, Litin LB, Willett WC: **Comparison of measures of fatty acid intake by subcutaneous fat aspirate, food frequency questionnaire, and diet records in a free-living population of US men**. *Am J Epidemiol* 1992, **135**(4):418-427.

3. Ma J, Folsom AR, Shahar E, Eckfeldt JH: **Plasma fatty acid composition as an indicator of habitual dietary fat intake in middle-aged adults. The Atherosclerosis Risk in Communities (ARIC) Study Investigators**. *Am J Clin Nutr* 1995, **62**(3):564-571.

4. Marckmann P, Lassen A, Haraldsdottir J, Sandstrom B: **Biomarkers of habitual fish intake in adipose tissue**. *Am J Clin Nutr* 1995, **62**(5):956-959.

5. Prisco D, Filippini M, Francalanci I, Paniccia R, Gensini GF, Abbate K, Neri Serneri GG: **Effect of n-3 polyunsaturated fatty acid intake on phospholipid fatty acid composition in plasma and erythrocytes**. *Am J Clin Nutr* 1996, **63**(6):925-932.

6. Garland M, Sacks FM, Colditz GA, Rimm EB, Sampson LA, Willett WC, Hunter DJ: **The relation between dietary intake and adipose tissue composition of selected fatty acids in US women**. *Am J Clin Nutr* 1998, **67**(1):25-30.

7. Baylin A, Kabagambe EK, Siles X, Campos H: **Adipose tissue biomarkers of fatty acid intake**. *Am J Clin Nutr* 2002, **76**(4):750-757.

8. Ritter-Gooder PK, Lewis NM, Heidal KB, Eskridge KM: **Validity and reliability of a quantitative food frequency questionnaire measuring n-3 fatty acid intakes in cardiac patients in the Midwest: a validation pilot study**. *J Am Diet Assoc* 2006, **106**(8):1251-1255.

9. Holmes MD, Powell IJ, Campos H, Stampfer MJ, Giovannucci EL, Willett WC: **Validation of a food frequency questionnaire measurement of selected nutrients using biological markers in African-American men**. *Eur J Clin Nutr* 2007, **61**(11):1328-1336.

10. Arsenault LN, Matthan N, Scott TM, Dallal G, Lichtenstein AH, Folstein MF, Rosenberg I, Tucker KL: **Validity of estimated dietary eicosapentaenoic acid and docosahexaenoic acid intakes determined by interviewer-administered food frequency questionnaire among older adults with mild-to-moderate cognitive impairment or dementia**. *Am J Epidemiol* 2009, **170**(1):95-103.
